# Evaluation of the Gastrointestinal Tract as Potential Route of Primary Polyomavirus Infection in Mice

**DOI:** 10.1371/journal.pone.0150786

**Published:** 2016-03-03

**Authors:** Gang Huang, Gang Zeng, Yuchen Huang, Bala Ramaswami, Parmjeet Randhawa

**Affiliations:** 1 Department of Organ Transplantation, The First Affiliated Hospital, Sun Yat-sen University, Guangzhou, China, 510080; 2 Department of Pathology, University of Pittsburgh, School Of Medicine, Pittsburgh, Pennsylvania, United States of America, 15261; 3 Department of Surgery, University of Pittsburgh, School Of Medicine, Pittsburgh, Pennsylvania, United States of America, 15261; University of Alberta, CANADA

## Abstract

**Background:**

Detection of Polyomavirus (PyV) DNA in metropolitan rivers worldwide has led to the suggestion that primary viral infection can occur by the oral route. The aim of this study was to test this notion experimentally.

**Methods:**

Mouse PyV (MPyV) was used to infect C57BL/6J mice by the nasal or intragastric route. Viral load kinetics was studied 3, 7, 10, 14, 21 and 28 days post-infection (dpi) using quantitative PCR.

**Results:**

Following nasal infection, MPyV DNA was readily detected in many organs including lung, heart, aorta, colon, and stool with viral loads in the range of 10^3^–10^6^ copies/mg wet weight that peaked 7–10 dpi. Complete viral clearance occurred in the serum and kidney by 28 dpi, while clearance in other organs was partial with a 10–100 fold decrease in viral load. In contrast, following intragastric infection peak detection of PyV was delayed to 21 dpi, and viral loads were up to 3 logs lower. There was no detectable virus in the heart, colon, or stool.

**Conclusions:**

The intragastric route of MPyV infection is successful, not as efficacious as the respiratory route, and associated with delayed viral dissemination as well as a lower peak MPyV load in individual organs.

## Introduction

The number of known human and animal species in the family Polyomaviridae has increased from less than five at the beginning of the twenty first century to approximately thirty in 2015 [1]. Several species have already been associated with disease in man including polyomavirus BK, JC, SV40, Merkel cell polyomavirus, human polyomavirus 7, 10, and 12 [2]. Clinical syndromes attributed to these viruses include post-transplant nephropathy, hemorrhagic cystitis, progressive multifocal encephalopathy, trichodysplasia spinosum, WHIM syndrome, pruritic skin rash, and gastro-esophageal disease. Merkel cell polyomavirus is now accepted as a cause of skin cancer, while polyomavirus BK, JC, and SV40 have been linked to urothelial carcinoma, glioblastoma, mesothelioma, and osteogenic sarcoma [3, 4].

Given the obvious medical importance of the polyomaviruses, a greater understanding of viral epidemiology is needed to devise suitable control strategies at the population level. Surprisingly little hard data is currently available on the actual mode of transmission of these viruses. There is a general belief that initial infection occurs by the respiratory route during childhood. This infection is typically asymptomatic and results in viral latency. Subsequent viral reactivation occurs when the host is immunosuppressed and results in clinical disease. In recent years polyomavirus BK, JC, and Merkel cell virus DNA have been detected in fresh river water, sewage, seawater, and human fecal samples taken from several different geographical locations worldwide [5–8]. These observations have raised the strong possibility that waterborne transmission is a key element in the natural biology of polyomavirus infection.

With respect to the biological plausibility of water borne transmission, it can be noted that the virus is relatively stable in raw sewage, since 90% reduction in virus concentration takes 53.6 days in samples maintained at 20°C and pH 5.0 or higher [8]. Moreover, the virus survives DNAase treatment suggesting that it may be present in the form of intact infectious particles with a complete protein capsid. Water treatment procedures implemented by municipalities reduce but do not totally eliminate this virus in human sewage [9].

In this study we have infected naïve mice by the intragastric route and followed viral replication kinetics in multiple organs using quantitative PCR. The results obtained are compared with a second group of mice infected by the nasal route, which is currently believed to be the natural mode of primary polyomavirus infection. To our knowledge, the natural history of polyomavirus infection following intragastric infection has not yet been previously studied.

## Methods

### Animals

All experiments were performed using groups of four 4–6 week, 15–20 gram, polyomavirus seronegative male C57/BL6 mice (Jackson Laboratories). Animals were housed in microisolator caging in a primate facility to prevent MPyV infection of other mouse colonies maintained at our institution. This virus is highly species-specific and not known to infect animals other than mice. Animals were monitored daily for overall activity level, lethargy, hair loss, respiratory distress, ruffled fir, skin rash, diarrhea, and bleeding. Transient respiratory distress was observed after intra-nasal inoculation but the animals resumed normal activity soon after. One animal in the nasal group was found dead 6 days post-infection during routine monitoring. Autopsy did not reveal an anatomic cause of death. At the end of the observation period animals were euthanized using carbon dioxide inhalation followed by cervical dislocation.

### Ethical Statement

The Institutional Animal Care and Use Committee (IACUC) of the University of Pittsburgh approved this study. All procedures were conducted in compliance with the IACUC guidelines in place at the University of Pittsburgh for pathogens designated as Biosafety Level II.

### Preparation of infectious viral inoculum

Mouse polyomavirus LID-1 strain (ATCC VR-252) was propagated in NIH 3T3 Cells using twenty T175 flasks in DMEM tissue culture medium with 10% FBS. Confluent cells were harvested using trypsin, lysed by freeze thawing and the lysate used to prepare mouse polyomavirus virions using sucrose and cesium chloride gradients using a published method [10]. The virus preparation was titered using quantitative PCR.

### Infection procedures

Animals were anesthetized using ketamine HCL 0.1mg/g (Bioriche Teoranta Inverin, Co. Galway, Ireland) and xylazine 0.01 mg/g (Lloyd Laboratories, Shenandoah, Iowa). Intragastric infection was effected by a 20 gauge, 3.8 cm, 2.4 mm tip, feeding tube (Fisher Scientific, cat# FNC 20–1.5, Pittsburgh, PA) which delivered 1x10^9^ genomic equivalents of MPyV suspended in 125 μl 0.9% saline. For intranasal infection the same dose of virus was suspended in 25 μl volume. After the animals were anesthetized, inoculum was delivered in 5 μl aliquots alternating between the right and left nostrils, with a 10 min waiting period between successive doses. A transient increase in respiratory rate was observed during this maneuver [11]. The dose of virus infected was determined empirically. In preliminary experiments, injection of 1x10^6^ to 1x 10^8^ genomic equivalents did not result in successful infection. Historically investigators have most often used 1E+06 to 1E+07 plaque forming units (PFU) of virus to infect mice, with 1 PFU = 100–1000 genomic equivalents as measured by PCR.

### Specimen collection

Urine, blood, and solid organs were collected 3, 7, 10, 14, 21 and 28 days after infection. Urine collection was facilitated by injecting 0.5–1.0 ml normal saline subcutaneously and waiting 30 mins for diuresis. This allowed direct aspiration of 10–500μl of urine directly from the urinary bladder. Blood was taken from the heart using 3 ml syringes with 21 gauge needles and 0.5ml serum separator tubes (Becton Dickinson cat# MC0530, Franklin Lakes, NJ). Animals were then sacrificed using carbon dioxide inhalation followed by cervical dislocation. Organs were harvested and immediately frozen at 70C after representative tissue had been saved in formalin for histologic studies.

### Amplification of viral DNA

DNA was extracted using the QIAamp Fast DNA Stool Mini Kit (Cat#51604), the QIAamp DNA Mini Kit for serum and urine (Cat#51306), or the Qiagen DNeasy Blood &Tissue Kit (Cat# 69506) for all other organs. Viral DNA amplification utilized a TaqMan quantitative PCR reaction performed in an ABI Prism 7500 Real Time PCR System. Sequence Detector (ABI, Foster City, CA). The following oligonucleotide sequences derived from the mouse polyomavirus A2 Strain large T antigen gene were synthesized for the MPyV PCR reaction [12]:

Forward Primer: 5’-CTTCTGAGCAACCCGACCTATT -3’

Reverse Primer: 5’-TCTTGGTCGCTTTCTGGATACAG-3’

PCR amplification reactions were set up with the reaction mixture containing 5 μl template DNA, 0.8μM forward primer, 0.7μM reverse primer, in 10% SyBrGreen, 1.5 mM MgCl2, 0.625 mM dNTP, 0.5 U AmpliTaq and 0.2U AmpErase (Applied Biosystems, Foster City, California) in a final volume of 20 ul. The PCR conditions included incubation at 50°C for 2 min, followed by a first denaturation step of 10 minutes at 95°C, then 40 cycles of 95°C for 15 seconds (denaturation), and 60°C for 1 minute (reannealing and extension).

### Quantitation of viral genomic load

Each real time PCR reaction included a standard curve derived from a 8.94 Kb plasmid containing the full length 5.8Kb MPyV genome in the pxf3 vector. The threshold cycle (Ct) for viral DNA copy numbers ranging from 10^1^ to 10^7^ was used to calculate viral copy numbers in each sample of interest. Samples with Ct higher than the lowest point of the standard curve were considered negative for viral DNA. The detection limit of the assay was 10 viral genomic equivalents per reaction, which corresponded to a mean sensitivity of 2578 copies/ml for urine and 1922 copies/ml for serum.

### Histologic examination

Representative samples of lungs, liver, kidney, spleen, and urinary bladder, were examined for parenchymal injury and viral cytopathic effect using standard histologic techniques.

## Results

### Infection by the nasal route

**Fig 1:**
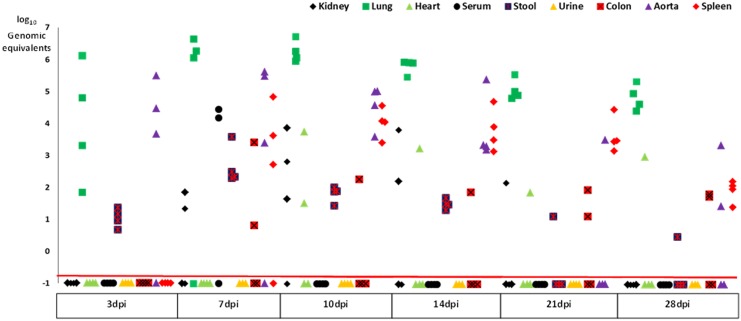
Dot plot of organ-specific viral loads in mice infected by the nasal route expressed as log_10_ viral genomic equivalents/mg wet weight for solid organs and copies/mL for body fluids. Samples plotted below the horizontal orange line had no detectable viral DNA.

Virus was first detected in lung, aorta and stool on day 3. Infection was abolished if the viral inoculum was boiled for 10 mins prior to infection. This shows that polyomavirus amplification from the lung on day 3 did not merely represent aspiration of the infectious inoculum. Initial detection of virus in the lung, aorta and stool was followed by detection in the spleen, colon, kidney, and serum on day 7, and heart on day 10 (Fig 1, Table 1). With respect to organ-specific viral replication kinetics, peak viral load occurred on day 7 for aorta, stool, and colon, but on day 10 for lung, kidney, and heart.

**Table 1:** Viral Loads Determined by Real Time Quantitative PCR after Nasal or Intragastric Infection (expressed as genomic equivalents/mg wet weight for solid organs and stool and copies/mL for body fluids).

	3dpi^a^	7dpi	10dpi	14dpi	21dpi	28dpi
Kidney						
Nasal	0	2.41×10^1^±3.49×10^1^^b^	2.16×10^3^±3.83×10^3^	1.85×10^3^±3.58×10^3^	3.85×10^1^±7.70×10^1^	0
Intragastric	0	0	0	0	3.64×10^2^±7.27×10^2^	0
Lung						
Nasal	3.52×10^5^±6.60×10^5^	2.05×10^6^±2.08×10^6^	2.62×10^6^±2.35×10^6^	7.98×10^5^±3.08×10^5^	1.70×10^5^±1.56×10^5^	1.07×10^5^±9.85×10^4^
Intragastric	4.56×10^2^±3.67×10^2^	6.28×10^1^±8.52×10^1^	1.46×10^1^±2.06×10^1^	1.76×10^1^±3.52×10^1^	1.45×10^3^±1.16×10^3^	5.20×10^0^±1.04×10^1^
Heart						
Nasal	0	0	1.51×10^3^±2.99×10^3^	4.67×10^2^±9.33×10^2^	1.92×10^1^±3.84×10^1^	2.63×10^2^±5.26×10^2^
Intragastric	0	0	0	0	0	0
Aorta						
Nasal	8.54×10^4^±1.49×10^5^	1.98×10^5^±2.32×10^5^	6.65×10^4^±5.29×10^4^	7.09×10^4^±1.38×10^5^	8.57×10^2^±1.71×10^3^	6.09×10^2^±1.20×10^3^
Intragastric	0	0	0	0	2.02×10^2^±4.02×10^2^	0
Stool						
Nasal	1.27×10^1^±7.78×10^0^	1.19×10^3^±1.88×10^3^	7.33×10^1^±3.22×10^1^	3.50×10^1^±1.32×10^1^	3.36×10^0^±6.71×10^0^	7.83×10^−1^±1.56×10^0^
Intragastric	0	0	0	0	0	0
Colon						
Nasal	0	6.84×10^2^±1.36×10^3^	4.74×10^1^±9.47×10^1^	1.95×10^1^±3.90×10^1^	2.61×10^1^±4.37×10^1^	3.17×10^1^±3.70×10^1^
Intragastric	0	0	0	0	0	0
Spleen						
Nasal	0	1.95×10^4^±3.57×10^4^	1.70×10^4^±1.60×10^4^	1.73×10^4^±2.55×10^4^	9.83×10^3^±1.44×10^4^	1.07×10^2^±6.13×10^1^
Intragastric	0	0	0	2.12×10^1^±4.25×10^1^	7.15×10^2^±1.41×10^3^	0
Urine						
Nasal	0	0	0	0	0	0
Intragastric	4.89×10^2^±9.79×10^2^	0	0	0	0	0
Serum						
Nasal	0	1.50×10^4^±1.77×10^4^	0	0	0	0
Intragastric	0	0	1.50×10^1^±1.77×10^1^	0	0	0

^a^ dpi, days post infection

^b^ Values are mean +/- sd.

Complete viral clearance occurred in the serum on day10, followed by kidney on day 28. Viral DNA remained detectable in all other organs on day 28 with actual viral loads being approximately 10% of peak levels in the lung, heart, colon, and stool, and 1% of the peak in the spleen and aorta. Serum MPyV DNA was seen at a single time point on day 7 (15,000 copies/ml). Urine viral load remained below the mean limit of detection which was 2578 copies/ml.

### Infection by the intragastric route

**Fig 2:**
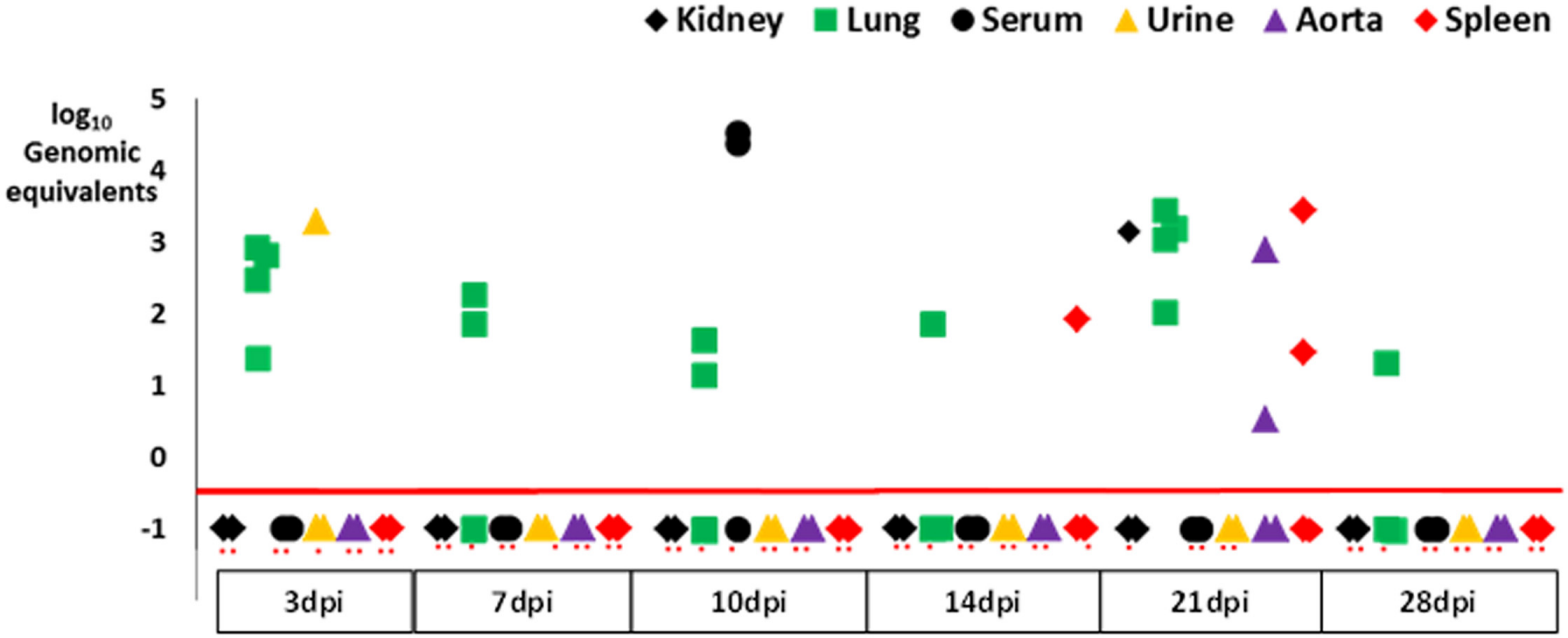
Dot plot of organ-specific viral loads in mice infected by the intragastric route expressed as log_10_ viral genomic equivalents/mg wet weight for solid organs and copies/mL for body fluids. Samples plotted below the horizontal red line had no detectable viral DNA. On the X-axis, red dots beneath the square, diamond, circle, and triangle organ symbols represent 2 mice each. Heart, stool, colon are not shown because all of these samples tested negative.

Mock infection was not considered necessary for animals infected by the gastrointestinal route since the stomach and colon tested negative at all time points. Thus, there was no concern that we were detecting residual inoculum in the samples analyzed. Virus was first detected at low levels in the lung and urine on day 3 (Table 1, Fig 2). This was followed by detection in serum on day 10, spleen on day 14, and the kidney as well as aorta on day 21. Amongst all the organs, the lung had the highest viral loads on all days evaluated. Viral load in the spleen was comparable to the lung on days 14 and 21. The largest number of organs infected was on day 21. Lung, aorta, and kidney viral loads were lower after intragastric than nasal infection, while stool tested negative polyomavirus DNA. By day 28, virus had been cleared from all organs other than the lung, where residual viral DNA constituted 1% of the peak level. Virus was detectable in the urine of one mouse on day 3. Unlike polyomavirus infection in kidney transplant patients viremia did not occur concurrently with but rather followed peak viruria.

### Animal to animal variability

Following nasal infection, the lung was the only organ in which polyomavirus could be detected in all animals exposed to virus at each of the evaluated time points. Infection of all 4 animals at least one time point could be observed in the aorta, stool and spleen. In general, the number of positive organs was lower in groups of samples taken away from the time of peak viral load. This indicates lack of consistent detection in tissues with low viral burden. Even greater animal to animal variability in detection of viral DNA was observed with the intragastric route (Table 2). At many time points only one or two of the four animals exposed to polyomavirus had viral DNA detectable in the organ of interest. However, we believe infection with the intragastric route was successful for each animal since 4/4 lungs examined at day 3 and 21 tested positive. We did consider the possibility that the low frequency detection in animals infected by the intragastric route was a technical false positive. However, the PCR assay was subject to stringent quality control, and no viral DNA was ever detected in control non-infected animals.

**Table 2:** Frequency of Detectable Viral DNA in Different Organs after Groups of 4 mice were Infected by the Nasal or Intragastric Route.

	Infectious route^a^	3dpi^b^	7 dpi	10 dpi	14 dpi	21 dpi	28 dpi
Kidney	Nasal/intragastric	0/0	2/0	3/0	2/0	1/1	0/0
Lung	Nasal/intragastric	4/4	3/2	4/2	4/1	4/4	4/1
Heart	Nasal/intragastric	0/0	0/0	2/0	1/0	1/0	1/0
Aorta	Nasal/intragastric	3/0	3/0	4/0	4/0	1/2	2/0
Stool	Nasal/intragastric	4/0	4/0	4/0	4/0	1/0	1/0
Colon	Nasal/intragastric	0/0	2/0	1/0	1/0	2/0	2/0
Spleen	Nasal/intragastric	0/0	3/0	4/0	4/1	4/2	4/0
Urine	Nasal/intragastric	0/1	0/0	0/0	0/0	0/0	0/0
Serum	Nasal/intragastric	0/0	2/0	0/2	0/0	0/0	0/0

^a^ Groups of 4 mice were infected at each time point using the nasal or, intragastric route. The actual number of animals that tested positive in these two categories is indicated.

^b^ dpi, days post infection

### Histologic examination

Histologic examination of the kidneys showed no interstitial inflammation, tubulitis, or viral inclusions in the tubular epithelium. The spleen, liver, and lungs also showed no cytopathic effect in the tissues. The animals maintained their normal weight gain, and did not show any external manifestations of illness. Thus, all infections were subclinical.

## Discussion

Published experimental models of MPyV infection have utilized the intra-peritoneal, subcutaneous and nasal routes [12, 13]. An intra-renal route of infection has also been described in which virus inoculated into one kidney led to ipsilateral peri-renal infection and then spread to the contralateral side along the peritoneum. These studies demonstrate that infection passes thru three distinct phases: primary viral replication, viremia, and systemic infection. Previously described sites of primary viral replication include the nasal cavity, submandibular salivary gland, lungs, and the mucosa lining the stomach [14, 15]. The viremia phase begins immediately after primary replication and continues into the late stages of dissemination.

The biologic consequences of infection can be altered by the route of infection. Thus, intranasal infection of newborn mice leads to high viral load in the lungs, persistence of latent virus after clearance of primary infection, but typically no oncogenesis. In contrast, intraperitoneal infection, which is characterized by drainage of viral antigens into the thoracic duct and vena cava [16], does not lead to initial virus replication in the lungs. Viral clearance is followed by development of tumors in multiple organs [14]. The age of the infected animal is also an important determinant of viral load: higher viral titers are obtained in newborn mice compared to weanling mice [17].

In the experiments reported here, the kinetics of viral replication following nasal infection was generally comparable to what is reported in the literature. Peak viremia coincided with peak viral loads in the spleen, aorta, and lung. It is surprising there was no detectable virus in the colon or stool at any time point following intragastric infection, including day 3, which was our earliest day of sacrifice. To provide reassurance that our stool PCR assay was capable of detecting small amounts of viral DNA, we then collected stool pellets from three different animal cages 24 hours after intragastric infection. Polyomavirus DNA derived from the infectious inoculum was detectable in these samples at a mean concentration of 1.90 x 10^2^ copies per mg.

The intragastric route of infection is not previously described in the literature. We found infection to have a delayed time course with much lower peak viral loads than could be achieved by the nasal route. This is likely because ingested virus is subjected to unfavorable pH and enzymatic digestion, which lower the effective size of the virus dose delivered to the animals.

The variability in the number of animals infected at any given time point is not readily explained. Genetic differences in innate and cell mediated immunity are unlikely in these inbred mice, although a potential role of epigenetic immunoregulatory mechanisms cannot be completely dismissed. The animal supplier confirmed that only polyomavirus seronegative animals are shipped to the customers. The performance of the PCR assay for viral DNA was comparable irrespective of route of infection, but only the intragastric routes, led to inconsistent viral loads, suggesting that route of infection was the critical factor. It is pertinent to note that the polyomavirus viral capsid is acid sensitive [8], and we did not measure gastric juice pH, which can show considerable meal related diurnal variation.

Replication of MPyV in the pulmonary system is not surprising if the initial infectious inoculum is delivered in the nostrils from where it can be inhaled into the lungs. However, pulmonary infection was also seen after administration of virus by the intragastric route. This may have been the consequence of viremia resulting from systemic viral replication in other organs later in the course of disease. With respect to the human counterpart of polyomavirus infection in the lung, well documented cases of polyomavirus BK pneumonitis have been documented in patients with chronic lymphocytic leukemia [18], AIDS [19], and hematopoietic stem cell transplantation [20].

Polyomavirus replication in the urogenital tract is of particular interest given its relevance to renal allograft nephropathy, which is the most common polyomavirus associated disease in man. Following nasal infection, viral DNA in the kidney was first detected on day 7; peak viral load was observed on days 10 & 14, followed by viral clearance on day 28. No virus could be detected in the urine at any time point with an assay that had a detection limit 2578 copies/ml. Following intragastric infection, detection of viral DNA in the kidney was delayed to day 21 and urinary virus was only detected on day 3. Our inability to detect viral DNA in the kidney of any animal after 28 dpi is at variance with some prior studies that indicate long-term virus persistence in the kidney. This somewhat unexpected finding may relate to differences in animal strain, age, virus strain, and virus detection methods. Individual mice with apparent resistance to kidney infection have been described by other investigators [17, 21–22]. Animal to animal and experiment to experiment variability in detection of urinary viral DNA has been noted in one prior study of newborn Swiss mice, and attributed to modulation of infection by antibody mediated immunity [17]. In general, the urogenital tract does not appear to be a very permissive site for MPyV, unless chemical or ischemic injury makes the cellular milieu more favorable for viral replication[23].

Polyomavirus infection of the cardiovascular system is not a common problem in clinical practice. In our experimental model, infection of the heart was detectable only after nasal of infection. Peak myocardial viral DNA content was observed after clearance of viremia, suggesting that involvement of the heart occurred in the late systemic phase of viral replication. Polyomavirus replication in the heart has also been noted by other investigators in mice and suckling hamsters [17, 24, 25]. In contrast to the heart, aortic infection was seen after both nasal and intragastric routes. Following nasal infection, aortic viral load kinetics was generally parallel to the lung and stool. Maximal viral burden was seen before peak serum viremia. However, following intragastric inoculation infection of the aorta was delayed, as it was in other organs. MPyV replication in large arteries has been described before in mice [1, 26]. Latent infection of the aorta has been recognized for three different human polyomaviruses, namely BK virus, JC virus and Merkel cell polyomavirus [27, 28]. Blood vessels other than the aorta also seem susceptible to virus, as is brought out by the case report of a kidney transplant patient with BK polyomavirus associated vasculopathy in the skeletal muscle complicated by a capillary leak syndrome [29].

The pattern of viral detection in the gastrointestinal tract after nasal infection calls for comment. Viral loads in the colon and stool were generally comparable. The stool viral load showed a close temporal correlation with the viral load in the aorta and lung. Detection of viral DNA in the stool preceded detection in the colon, yet viral clearance occurred first in the colon. This indicates that virus detected in the stool is not entirely derived from the colon, and that there may be additional contributions from other parts of the gastrointestinal tract including possibly the liver and pancreas. Polyomavirus infection of the colon has been previously documented by whole mouse hybridization [14, 16]. With regard to humans, polyomavirus SV40 has been detected in the stool of hospitalized children and accidental recipients of virus contaminated polio vaccines [30, 31]. There is also a case report of a kidney transplant patient who presented with diarrhea, colonic ulcers, inclusion bodies, and a positive PCR test for serum BKV DNA [32].

In conclusion, this study documents for the first time that systemic polyomavirus infection can result from intragastric inoculation. Compared to nasal infection, infection by the intragastric route is less efficient, follows a slower time course, and is associated with a lower peak viral load. It is also shown that systemic infection leads to viral excretion in the colon and this observation provides a potential, albeit not very efficient, mechanism for feco-oral transmission in the polyomavirus life cycle.

## Abbreviations

PyV: Polyomavirus
dpi: days post- infection
MPyV: Mouse PyV
IACUC: Institutional Animal Care and Use Committee
